# The Language Network Is Recruited but Not Required for Nonverbal Event Semantics

**DOI:** 10.1162/nol_a_00030

**Published:** 2021-03-17

**Authors:** Anna A. Ivanova, Zachary Mineroff, Vitor Zimmerer, Nancy Kanwisher, Rosemary Varley, Evelina Fedorenko

**Affiliations:** Department of Brain and Cognitive Sciences, Massachusetts Institute of Technology, Cambridge, MA, USA; McGovern Institute for Brain Research, Massachusetts Institute of Technology, Cambridge, MA, USA; Division of Psychology and Language Sciences, University College London, London, UK

**Keywords:** fMRI, aphasia, events, semantics, thematic roles, language and thought

## Abstract

The ability to combine individual concepts of objects, properties, and actions into complex representations of the world is often associated with language. Yet combinatorial event-level representations can also be constructed from nonverbal input, such as visual scenes. Here, we test whether the language network in the human brain is involved in and necessary for semantic processing of events presented nonverbally. In Experiment 1, we scanned participants with fMRI while they performed a semantic plausibility judgment task versus a difficult perceptual control task on sentences and line drawings that describe/depict simple agent–patient interactions. We found that the language network responded robustly during the semantic task performed on both sentences and pictures (although its response to sentences was stronger). Thus, language regions in healthy adults are engaged during a semantic task performed on pictorial depictions of events. But is this engagement necessary? In Experiment 2, we tested two individuals with global aphasia, who have sustained massive damage to perisylvian language areas and display severe language difficulties, against a group of age-matched control participants. Individuals with aphasia were severely impaired on the task of matching sentences to pictures. However, they performed close to controls in assessing the plausibility of pictorial depictions of agent–patient interactions. Overall, our results indicate that the left frontotemporal language network is recruited but not necessary for semantic processing of nonverbally presented events.

## INTRODUCTION

Many thinkers have argued for an intimate relationship between language and thought, in fields as diverse as philosophy (Carruthers, 2002; Davidson, 1975; Wittgenstein, 1961), psychology (Sokolov, 1972; Vygotsky, 2012; Watson, 1920), linguistics (Berwick & Chomsky, 2016; Bickerton, 1990; Chomsky, 2007; Hinzen, 2013; Jackendoff, 1996), and artificial intelligence (Brown et al., 2020; Goldstein & Papert, 1977; Turing, 1950; Winograd, 1976). According to such accounts, language enables us to access our vast knowledge of objects, properties, and actions—often referred to as *semantic knowledge*—and flexibly combine individual semantic units to produce complex situation-specific representations called *thoughts*. The hypothesis that language is critical for thought crucially depends on whether or not language is essential for combinatorial *semantic processing*: If we can access and combine individual concepts in the absence of language, that would constitute evidence against the necessity of language in forming novel thoughts. Here, we test the link between language and thought by examining the role of the language network in a nonverbal combinatorial semantic task.

Recent evidence from neuroscience suggests that language processing is largely distinct from other aspects of cognition (Fedorenko & Blank, 2020; Fedorenko & Varley, 2016). A network of left-lateralized frontal and temporal brain regions (here referred to as *the language network*) has been found to respond to written/spoken/signed words and sentences, but not to mental arithmetic, music perception, executive function tasks, action/gesture perception, or computer programming (Amalric & Dehaene, 2019; X. Chen et al., 2021; Fedorenko et al., 2011; Ivanova et al., 2020; Jouravlev et al., 2019; Liu et al., 2020; MacSweeney et al., 2002; Monti et al., 2009, 2012; Pritchett et al., 2018). Similarly, investigations of patients with profound disruption of language capacity (*global aphasia*) have shown that some of these individuals can solve arithmetic and logic problems, appreciate and create music, and think about others’ thoughts in spite of their language impairment (Basso & Capitani, 1985; Luria et al., 1965; Varley et al., 2005; Varley & Siegal, 2000), providing converging evidence that language is subserved by domain-specific cognitive mechanisms.

Despite this significant progress in dissociating linguistic and nonlinguistic processing, the role of the language network in nonverbal semantics remains unclear. Semantics is often considered to be an integral part of linguistic processing (Altshuler et al., 2019; Binder et al., 2009; Fillmore, 2006; Milberg & Blumstein, 1981; Pinker & Levin, 1991; Talmy, 2000): Each content word is linked to an underlying semantic representation (*lexical semantics*), which then combine to form phrase- and sentence-level meanings (*combinatorial semantics*). This tight integration between language and semantics suggests that the frontal and temporal language regions may play an important role in storing and processing semantic information (see Hasson et al., 2015, for general arguments against the separation of storage and processing/computation in the brain). However, many semantic representations can also be activated by nonverbal input (e.g., the concept CAT can be evoked not only by the word “cat,” but also by a picture or the sight of a cat), suggesting that language does not necessarily have a privileged role in semantic processing. In this work, we ask whether the frontotemporal language network supports semantic processing for both verbal and nonverbal stimuli or whether it is only engaged in the semantic processing of *verbal* input.

A large body of work has aimed to address the role of the language network in nonverbal semantics; however, different sources of evidence have produced conflicting results. Neuroimaging studies that explicitly compared verbal and nonverbal semantic processing of objects (e.g., Devereux et al., 2013; Fairhall & Caramazza, 2013; Handjaras et al., 2017; Shinkareva et al., 2011; Vandenberghe et al., 1996; Visser et al., 2012), actions (e.g., Wurm & Caramazza, 2019), and events (Baldassano et al., 2018; Hu et al., 2019; Jouen et al., 2015; Thierry & Price, 2006) often report overlapping activation in left-lateralized frontal and temporal areas, which may reflect the engagement of the language network. In contrast, neuropsychology studies have often reported dissociations between linguistic and semantic deficits in patients with aphasia (e.g., Antonucci & Reilly, 2008; Chertkow et al., 1997; Dickey & Warren, 2015; Jefferies & Lambon Ralph, 2006; Saygın et al., 2004; cf. Saygın et al., 2003), suggesting that verbal and nonverbal semantic processes rely on distinct neural circuits. Both groups of studies have limitations that make it difficult to reconcile their findings. The neuroimaging studies have typically relied on group analyses—an approach known to overestimate overlap in cases of nearby functionally distinct areas (Nieto-Castañón & Fedorenko, 2012)—and/or do not report effect sizes, which are critical for interpreting the functional profiles of the regions in question (a region that responds similarly strongly to verbal and nonverbal semantic tasks plausibly supports computations that are different from a region that responds to both, but shows a two to three times stronger response to verbal semantics; see, e.g., G. Chen et al., 2017, for discussion). Meanwhile, the aphasia studies have typically investigated cases where only some of the language regions were damaged, leaving open the possibility that the intact portions of the language network were still contributing to nonverbal semantic processing. Further, neuroimaging and aphasia studies typically rely on different experimental paradigms, making it challenging to directly compare their results.

It should also be noted that few neuropsychological studies (with the exception of Dresang et al., 2019; Marshall et al., 1993) have investigated the processing of verbal and nonverbal events (as opposed to individual objects or actions). Constructing event-level mental representations requires object and action processing but is not reducible to them (Dresang et al., 2019) and therefore may engage additional cognitive operations. In particular, to understand an *event*, we must identify *relations* between participating entities and assign them *thematic roles* (Estes et al., 2011). This process of identifying who did what to whom has traditionally been considered a hallmark of the language system (Fillmore, 1968; Gruber, 1965). Thus, if any aspect of semantic processing requires language, event understanding would seem to be one of the strongest candidates.

Event processing has perhaps been most extensively investigated in EEG research, where a number of studies have reported that semantic violations in visually presented scenes/events evoke the N400 response, a marker of semantic processing (Coco et al., 2020; Cohn, 2020; Jouen et al., 2019; Proverbio & Riva, 2009; Sitnikova et al., 2008; Võ & Wolfe, 2013; West & Holcomb, 2002; see Kutas & Federmeier, 2011, for a review), similarly to semantic violations in sentences, where the N400 component was originally discovered (Kutas & Hillyard, 1980). The EEG results have been taken to suggest that linguistic and visual semantic processing rely on a shared mechanism. However, because the neural generators of the N400 remain debated (Lau et al., 2008, 2016; Matsumoto et al., 2005; Zhu et al., 2019), this evidence does not definitively demonstrate the involvement of the language network in visual event processing.

Here, we synergistically combine neuroimaging and neuropsychological evidence to ask whether the language network is engaged during and/or is necessary for nonverbal event semantics. We focus on the understanding of agent–patient relations (“who did what to whom”) in visually presented scenes. Identification of thematic relations is critical to understanding and generating sentences (Carlson & Tanenhaus, 1988; Fillmore, 2002; Jackendoff, 1987), but *agent* and *patient* are not exclusively linguistic notions: They likely constitute part of humans’ *core knowledge* (Rissman & Majid, 2019; Spelke & Kinzler, 2007; Strickland, 2017; L. Wagner & Lakusta, 2009) and are integral to visual event processing (Cohn & Paczynski, 2013; Hafri et al., 2018). Investigating the role of the language network in processing agent–patient relations therefore constitutes an important test of the relationship between language and combinatorial event semantics.

We used two kinds of evidence in our study: (1) fMRI in neurotypical participants, and (2) behavioral data from two individuals with global aphasia and a group of age-matched healthy controls. All participants were asked to evaluate the *plausibility of events*, presented either as sentences (neurotypicals only) or as pictures. To ensure that participants could not rely on low-level visual cues when evaluating picture plausibility, we used line drawings rather than photographs. The line drawings were highly controlled: Each picture pair depicted two animate participants engaged in a certain interaction, but the participants’ roles in this interaction were either plausible (e.g., a cop arresting a criminal) or implausible (e.g., a criminal arresting a cop). This manipulation allowed us to ensure that participants could not infer picture plausibility based solely on the attributes of a single participant; rather, they had to evaluate the event as a whole.

To foreshadow our results, we find that language-responsive brain areas in neurotypical participants respond during the plausibility task for both sentences and pictures (although the responses are lower for pictures). However, individuals with global aphasia, who sustained severe damage to language areas, perform well on the picture plausibility task, suggesting that the language network is not required for constructing combinatorial representations of visually depicted events.

## MATERIALS AND METHODS

### Experiment 1: Is the Language Network Active During a Nonverbal Event Semantics Task?

#### Overview

**Figure 1.**
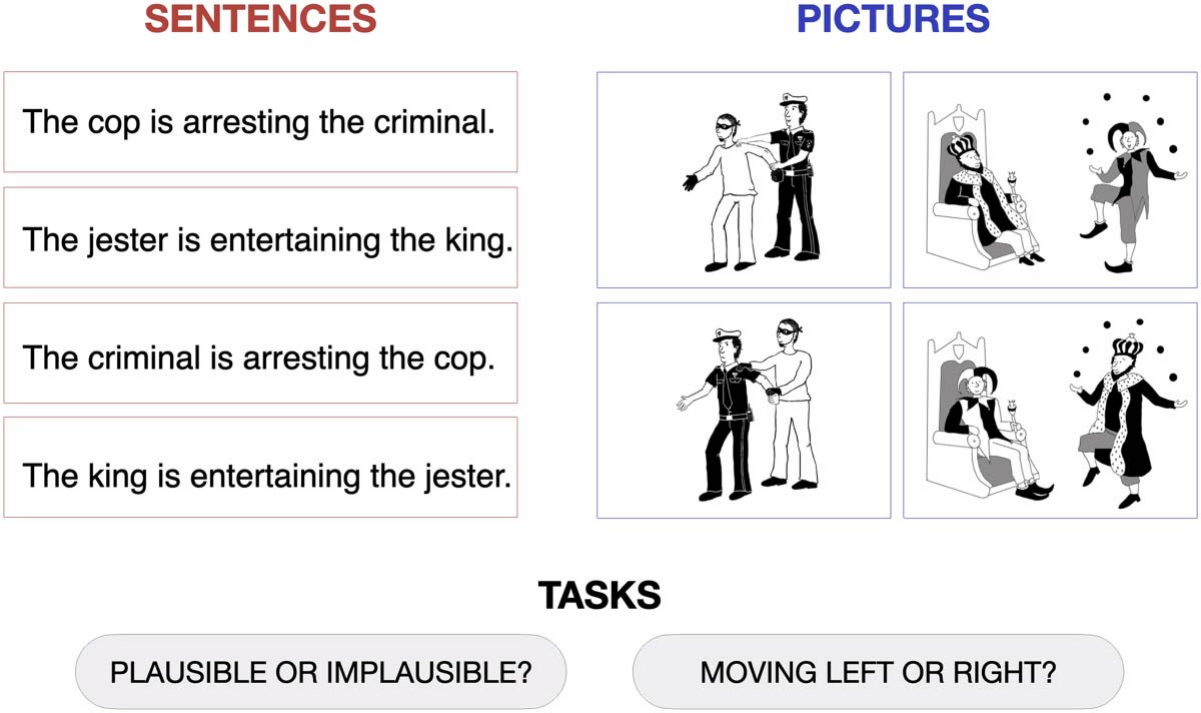
Sample stimuli used in the experiment. For both sentences and pictures, participants were required to perform either a semantic plausibility judgment task (“Plausible or implausible?”) or a control perceptual task (“Moving left or right?”). The full set of materials is available at https://osf.io/gsudr/.

In the first experiment, we presented neurotypical participants with sentences and pictures describing/depicting agent–patient interactions that were either plausible or implausible (Figure 1), while the participants were undergoing an fMRI scan. Participants performed a semantic judgment task on the sentences and pictures, as well as a difficulty-matched low-level perceptual control task on the same stimuli, in a 2 × 2 blocked design. In separate blocks, participants were instructed to indicate either (i) whether the stimulus was plausible or implausible (the semantic task) or (ii) whether the stimulus was moving to the left or right (the perceptual task). The language regions in each participant were identified using a separate functional language localizer task (sentences > nonwords contrast; Fedorenko et al., 2010). We then measured the response of those regions to sentences and pictures during the semantic and perceptual tasks.

#### Participants

Twenty-four participants took part in the fMRI experiment (11 female, mean age = 25 years, *SD* = 5.2). The participants were recruited from MIT and the surrounding Cambridge/Boston, MA, community and paid for their participation. All were native speakers of English, had normal hearing and vision, and had no history of language impairment. All were right-handed (as assessed by Oldfield’s, 1971, handedness questionnaire, or self-report). Two participants had low behavioral accuracy scores (<60%), and one had right-lateralized language regions (as evaluated by the language localizer task; see below); they were excluded from the analyses, which were therefore based on data from 21 participants. The protocol for the study was approved by MIT’s Committee on the Use of Humans as Experimental Subjects (COUHES). All participants gave written informed consent in accordance with protocol requirements.

#### Design, materials, and procedure

All participants completed a language localizer task aimed at identifying language-responsive brain regions (Fedorenko et al., 2010) and the critical picture/sentence plausibility task.

The localizer task was conducted in order to identify brain regions within individual participants that selectively respond to language stimuli. During the task, participants read sentences (e.g., NOBODY COULD HAVE PREDICTED THE EARTHQUAKE IN THIS PART OF THE COUNTRY) and lists of unconnected, pronounceable nonwords (e.g., U BIZBY ACWORRILY MIDARAL MAPE LAS POME U TRINT WEPS WIBRON PUZ) in a blocked design. Each stimulus consisted of twelve words/nonwords. For details of how the language materials were constructed, see Fedorenko et al. (2010). The materials are available at https://evlab.mit.edu/funcloc/. The sentences > nonword-lists contrast has been previously shown to reliably activate left-lateralized frontotemporal language processing regions and to be robust to changes in the materials, task, and modality of presentation (Fedorenko et al., 2010; Mahowald & Fedorenko, 2016; Scott et al., 2017). Stimuli were presented in the center of the screen, one word/nonword at a time, at the rate of 450 ms per word/nonword. Each stimulus was preceded by a 100 ms blank screen and followed by a 400 ms screen showing a picture of a finger pressing a button, and a blank screen for another 100 ms, for a total trial duration of 6 s. Participants were asked to press a button whenever they saw the picture of a finger pressing a button. This task was included to help participants stay alert and awake. Condition order was counterbalanced across runs. Experimental blocks lasted 18 s (with 3 trials per block), and fixation blocks lasted 14 s. Each run (consisting of 5 fixation blocks and 16 experimental blocks) lasted 358 s. Each participant completed 2 runs.

The picture plausibility task included two types of stimuli: (1) black-and-white line drawings depicting plausible and implausible agent–patient interactions (created by an artist for this study), and (2) simple sentences describing the same interactions. Sample stimuli are shown in Figure 1, and a full list of materials is available on this article’s website (https://osf.io/gsudr/). Forty plausible-implausible pairs of pictures, and forty plausible-implausible pairs of corresponding sentences were used. The full set of materials was divided into two lists, such that List 1 used plausible pictures and implausible sentences for odd-numbered items, and implausible pictures and plausible sentences for even-numbered items, and List 2 did the opposite. Thus, each list contained either a picture or a sentence version of any given event. Stimuli were presented in a blocked design (each block included either pictures or sentences) and were moving either to the right or to the left for the duration of stimulus presentation. At the beginning of each block, participants were told which task they would have to perform next: semantic or perceptual. The semantic task required them to indicate whether the depicted/described event is plausible or implausible by pressing one of two buttons. The perceptual task required them to indicate the direction of stimulus movement (right or left). To ensure that participants always perform the right task, a reminder about the task and the response buttons (“plausible=1/implausible=2” or “moving right=1/left=2”) was visible in the lower right-hand corner of the screen for the duration of the block. Each stimulus (a picture or a sentence) was presented for 1.5 s, with 0.5 s intervals between stimuli. Each block began with a 2 s instruction screen to indicate the task, and consisted of 10 trials, for a total duration of 22 s. Trials were presented with a constraint that the same response (plausible/implausible in the semantic condition, or right/left in the perceptual condition) did not occur more than 3 times in a row. Each run consisted of 3 fixation blocks and 8 experimental blocks (2 per condition: semantic task – pictures; semantic task – sentences; perceptual task – pictures; perceptual task – sentences) and lasted 242 s (4 min 2 s). The order of conditions was palindromic and varied across runs and participants. Each participant completed 2 runs.

#### fMRI data acquisition

Structural and functional data were collected on the whole-body, 3 Tesla, Siemens Trio scanner with a 32-channel head coil, at the Athinoula A. Martinos Imaging Center at the McGovern Institute for Brain Research at MIT. T1-weighted structural images were collected in 176 sagittal slices with 1 mm isotropic voxels (TR = 2,530 ms, TE = 3.48 ms). Functional, blood oxygenation level dependent (BOLD) data were acquired using an echo-planar imaging sequence (with a 90° flip angle and using generalized autocalibrating partial parallel acquisition [GRAPPA] with an acceleration factor of 2), with the following acquisition parameters: thirty-one 4-mm-thick near-axial slices acquired in the interleaved order (with 10% distance factor), 2.1 mm × 2.1 mm in-plane resolution, FoV in the phase encoding (A>>P) direction 200 mm and matrix size 96 mm × 96 mm, TR = 2,000 ms and TE = 30 ms. The first 10 s of each run were excluded to allow for steady state magnetization.

#### fMRI data preprocessing

MRI data were analyzed using SPM12 and custom MATLAB scripts (available in the form of an SPM toolbox from http://www.nitrc.org/projects/spm_ss). Each participant’s data were motion corrected and then normalized into a common brain space (the Montreal Neurological Institute [MNI] template) and resampled into 2 mm isotropic voxels. The data were then smoothed with a 4 mm FWHM Gaussian filter and high-pass filtered (at 200 s). Effects were estimated using a General Linear Model in which each experimental condition was modeled with a boxcar function (modeling entire blocks) convolved with the canonical hemodynamic response function.

#### Defining functional regions of interest

The critical analyses were restricted to individually defined language functional regions of interest (fROIs). These fROIs were defined using the Group-Constrained Subject-Specific approach (Fedorenko et al., 2010; Julian et al., 2012), where a set of spatial parcels is combined with each individual subject’s localizer activation map to constrain the definition of individual fROIs. The parcels mark the expected gross locations of activations for a given contrast based on prior work and are sufficiently large to encompass the extent of variability in the locations of individual activations. Here, we used a set of six parcels derived from a group-level probabilistic activation overlap map for the sentences > nonwords contrast in 220 participants. These parcels included two regions in the left inferior frontal gyrus (IFG, IFGorb), one in the left middle frontal gyrus (MFG), two in the left temporal lobe (AntTemp and PostTemp), and one extending into the angular gyrus (AngG). (The parcels are available at https://osf.io/gsudr/). Within each parcel, we selected the top 10% most responsive voxels, based on the *t* values for the sentences > nonwords contrast (see Figure 1 in Blank et al., 2014, or Figure 1 in Mahowald & Fedorenko, 2016, for sample fROIs). Individual-level fROIs defined in this way were then used for subsequent analyses that examined the behavior of the language network during the critical picture/sentence plausibility task.

#### Examining the functional response profiles of the language fROIs

For each language fROI in each participant, we averaged the responses across voxels to get a value for each of the four critical task conditions (semantic task on pictures, semantic task on sentences, perceptual task on pictures, perceptual task on sentences). We then ran a linear mixed-effect regression model with two fixed effects (stimulus type and task) and two random intercepts (participant and fROI). We used sum coding for both stimulus type and task. Planned follow-up comparisons examined response to sentences and pictures during the semantic task within each fROI; the results were FDR-corrected (Benjamini & Hochberg, 1995) for the number of regions. The formula used for the main mixed linear effects model was *EffectSize* ∼ *StimType* * *Task* + (*1*|*fROI*) + (*1*|*Participant*). The formula used for the follow-up comparisons was *EffectSize* ∼ *StimType* * *Task* + (*1*|*Participant*).

The analysis was run using the *lmer* function from the *lme4* R package (Bates et al., 2015); statistical significance of the effects was evaluated using the *lmerTest* package (Kuznetsova et al., 2017).

#### Behavioral analyses

To analyze differences in response times (RT) and accuracy across conditions, we ran a linear (for RT) and logistic (for accuracy) mixed effect regression model that aimed to mirror the structure of the mixed effect models in the neuroimaging analyses. Specifically, the behavioral models used task and stimulus type as fixed effects (with sum contrast coding) and participant and item as random intercepts. The formulae were *Accuracy*/*RT* ∼ StimType*Task + (*1*|*fROI*) + (*1*|*Participant*).

### Experiment 2: Is the Language Network Required for a Nonverbal Event Semantics Task?

#### Overview

In the second experiment, we examined two individuals with global aphasia, a disorder characterized by severe linguistic impairments, together with a group of age-matched controls. The participants performed two critical tasks: the picture plausibility judgment task (identical to the “picture, semantic” condition from Experiment 1) and the sentence–picture matching task based on the same set of pictures.

#### Participants

Two participants with global aphasia, S.A. and P.R., took part in the study. Both had large lesions that had damaged the left IFG, the inferior parietal lobe (supramarginal and angular gyri), and the superior temporal lobe. At the time of testing, they were 68 and 70 years old respectively. S.A. was 22 years 5 months post-onset of his neurological condition, and P.R. was 14 years 7 months post-onset. S.A. had a subdural empyema in the left sylvian fissure, with associated meningitis that led to a secondary vascular lesion in left middle cerebral artery territory. P.R. also had a vascular lesion in left middle cerebral artery territory.

Both participants were male, native English speakers, and did not present with visual impairments. S.A. was premorbidly right-handed; P.R. was premorbidly left-handed, but a left hemisphere lesion that resulted in profound aphasia indicated that he, like most left-handers, was left hemisphere dominant for language (Pujol et al., 1999). Both individuals were classified as severely agrammatic (Table 1), but their nonlinguistic cognitive skills were mostly spared (Table 2). They performed the semantic task and the sentence–picture matching task with a 7-month period between the two.

**Table 1.** Regression model terms for fROI-based statistical analyses

**ROI**	**Regression Term**	**Beta**	***p* value**
IFGorb	Intercept	0.33	0.104
	Stimulus (Sent>Pic)	0.34	0.215
	**Task (Sem>Perc)**	**1.25**	**<0.001**
	Stimulus:Task	0.54	0.283
IFG	**Intercept**	**1.33**	**<0.001**
	Stimulus (Sent>Pic)	0.27	0.259
	**Task (Sem>Perc)**	**1.12**	**<0.001**
	Stimulus:Task	0.57	0.28
MFG	**Intercept**	**0.98**	**0.002**
	Stimulus (Sent>Pic)	0.2	0.259
	**Task (Sem>Perc)**	**0.73**	**<0.001**
	Stimulus:Task	0.74	0.231
AntTemp	Intercept	0.22	0.104
	**Stimulus (Sent>Pic)**	**0.49**	**0.002**
	**Task (Sem>Perc)**	**0.6**	**<0.001**
	Stimulus:Task	0.41	0.24
PostTemp	**Intercept**	**0.5**	**<0.001**
	**Stimulus (Sent>Pic)**	**0.43**	**0.006**
	**Task (Sem>Perc)**	**0.68**	**<0.001**
	Stimulus:Task	0.44	0.24
AngG	**Intercept**	**1.13**	**0.002**
	Stimulus (Sent>Pic)	−0.35	0.215
	**Task (Sem>Perc)**	**1.16**	**<0.001**
	Stimulus:Task	−0.11	0.823

*Note*. The *p* values are FDR-corrected for the number of regions (*n* = 6). Significant terms are highlighted in bold. The fROI labels correspond to the approximate anatomical locations: IFGorb – the orbital portion of the left inferior frontal gyrus; IFG – left inferior frontal gyrus; MFG – left middle frontal gyrus; AntTemp – left anterior temporal cortex; PostTemp – left posterior temporal cortex; AngG – left angular gyrus.

**Table 2.** Results of linguistic assessments for participants with global aphasia

**Lexical Tests**	**Chance Score**	**S.A.**	**P.R.**
ADA spoken word to picture matching	16.5	60/66*	61/66*
ADA written word to picture matching	16.5	62/66*	66/66*
ADA spoken synonym matching	80	123/160*	113/160*
ADA written synonym matching	80	121/160*	145/160*
PALPA 54 spoken picture naming	n/a	0/60	0/60
PALPA 54 written picture naming	n/a	24/60	2/60

**Syntactic Tests**			
Comprehension of spoken reversible sentences	50	49/100	38/100
Comprehension of written reversible sentences	50	42/100	49/100
Written grammaticality judgments	20	26/40*	21/40

**Verbal Working Memory**			
PALPA 13-digit span (recognition)	n/a	3 items	4 items

*Note*. The tests were taken from the Action for Dysphasic Adults (ADA) Auditory Comprehension Battery (Franklin et al., 1992) and the Psycholinguistic Assessment of Language Processing in Aphasia (PALPA; Kay et al., 1992) or developed for the purpose of the study.

* Indicates above chance performance (*p* < 0.05).

We also tested two sets of neurotypical control participants, one for the semantic task and one for the language task. The semantic task control participants were 12 healthy participants (7 females) ranging in age from 58 to 78 years (mean age 65.5 years). The language task control participants were 12 healthy participants (5 females) ranging in age from 58 to 78 years (mean age 64.7 years). None of the healthy participants had a history of speech or language disorders, neurological diseases, or reading impairments. All were native English speakers and had normal, or corrected-to-normal, vision.

Participants undertook the experiments individually, in a quiet room. An experimenter was present throughout the testing session. The stimuli were presented on an Acer Extensa 5630G laptop, with the experiment built using DMDX (Forster & Forster, 2003). Ethics approval was granted by the UCL Research Ethics Committee (LC/2013/05). All participants provided informed consent prior to taking part in the study.

#### Semantic task: Picture plausibility judgments

The same picture stimuli were used as in Experiment 1 (see Figure 1), plus one additional plausible-implausible pair of pictures (which was omitted from the fMRI experiment to have a total number of stimuli be divisible by four, for the purposes of grouping materials into blocks and runs), for a total of 82 pictures (41 plausible-implausible pairs). Four of the 82 pictures were used as training items (see below).

The stimuli were divided into two sets, with an equal number of plausible and implausible pictures; each plausible-implausible pair was split across the two sets, to minimize repetition of the same event participants within a set. The order of the trials was randomized within each set, so that each participant saw the pictures in a different sequence. A self-timed break was placed between the two sets.

Prior to the experiment, participants were shown two pairs of pictures, which acted as training items. The pairs consisted of one plausible and one implausible event. They were given clear instructions to focus on the relationship between the two characters and assess whether they thought the interaction was plausible, in adherence with normal expectations, or implausible, at odds with expectations. They were asked to press a green tick (the left button on the mouse) if they thought the picture depicted a plausible event, and a red cross (the right button on the mouse) if they thought the picture depicted an implausible event. They were asked to do so as quickly and accurately as possible. The pictures appeared for a maximum of 8 s, with the interstimulus interval of 2 s. Accuracies and reaction times were recorded. Participants had the opportunity to ask any questions, and the instructions for participants with aphasia were supplemented by gestures to aid comprehension of the task. Participants had to indicate that they understood the task prior to starting.

#### Language task: Sentence to picture matching

The same 82 pictures were used as in the plausibility judgment experiment. In this task, a sentence was presented below each picture that either described the picture correctly (e.g., “the cop is arresting a criminal” for the first sample picture in Figure 1) or had the agent and patient switched (“the criminal is arresting the cop”). Simple active subject-verb-object sentences were used. Combining each picture with a matching and a mismatching sentence resulted in 164 trials in total.

For the control participants, the trials were split into two sets of 82, with an equal number of plausible and implausible pictures, as well as an equal number of matches and mismatches in each set. In order to avoid tiring the participants with aphasia, the experiment was administered across two testing sessions each consisting of two sets of 41 stimuli and occurring within the same week. For both groups, the order of the trials was randomized separately for each participant, and no pictures from the same pair (e.g., an event involving a cop and a criminal) appeared in a row. A self-timed break was placed between the two sets.

Prior to the experiment, participants were told that they would see a series of pictures with accompanying sentences, and their task was to decide whether the sentence matched the depicted event. They were asked to press a green tick (the left button on the mouse) if they thought the sentence matched the picture, and a red cross (the right button on the mouse) if they thought the sentence did not match the picture. They were asked to do so as quickly and accurately as possible. The picture/sentence combinations appeared for a maximum of 25 s, with the interstimulus interval of 2 s. Accuracies and reaction times were recorded. As in the critical task, participants had the opportunity to ask any questions, and the instructions for participants with aphasia were supplemented by gestures.

#### Data analysis

We used the exact binomial test to test whether patients’ performance on either task was significantly above chance, as well as the Crawford and Howell (1998) test for dissociation to compare patient performance relative to controls across the two tasks. We excluded all items with reaction times and/or accuracies outside 3 standard deviations of the control group mean (4 items for the semantic task and 11 items for the sentence–picture matching task).

#### Estimating the damage to the language network in patients with aphasia

In order to visualize the extent of the damage to the language network, we combined the available structural MRI of one patient with aphasia (P.R.) with a probabilistic activation overlap map of the language network. The map was created by overlaying thresholded individual activation maps for the language localizer contrast (sentences > nonwords, as described in Experiment 1) in 220 healthy participants. The maps were thresholded at the *p* < 0.001 whole-brain uncorrected level, binarized, and overlaid in the common space, so that each voxel contains information on the proportion of participants showing a significant language localizer effect (see Woolgar et al., 2018, for more details). The map can be downloaded from https://osf.io/gsudr/.

## RESULTS

### Experiment 1: Is the Language Network Active During a Nonverbal Event Semantics Task?

#### Behavioral results

All participants were engaged during the task: the overall response rate was 91.7% (sentence semantic 89.9%; sentence perceptual 91.6%; picture semantic 93.6%; picture perceptual 91.9%). Average response times were 1.27 s (*SD* = 0.46) for the semantic sentence task, 1.16 s (*SD* = 0.38) for the perceptual sentence task, 1.22 s (*SD* = 0.35) for the semantic picture task, and 1.19 (*SD* = 0.36) for the perceptual picture task. A linear mixed effect model with *task* and *stimulus type* as fixed effects and *participant* and *item number* as random intercepts showed a small main effect of task (semantic > perceptual; *β* = 0.06, *p* < 0.001), no main effect of stimulus type (*β* = 0.02, *p* = 0.287), and no interaction between task and stimulus type (*β* = 0.03, *p* = 0.359).

Average accuracies were 0.81 for the semantic sentence task, 0.79 for the perceptual sentence task, 0.75 for the semantic picture task, and 0.75 for the perceptual picture task. A logistic mixed effect model with the same structure as the linear RT model above showed no significant effects of either task (*β* = 0.09, *p* = 0.198) or stimulus type (*β* = 0.12, *p* = 0.101), and no interaction between them (*β* = 0.04, *p* = 0.759). Due to a technical error, accuracy data for 14 participants were only recorded for one of the two runs.

#### Neuroimaging results

**Figure 2.**
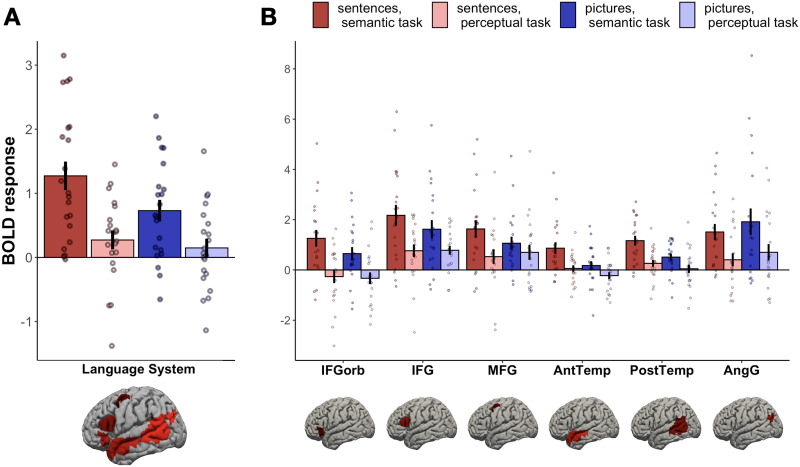
BOLD response during the four experimental conditions within (A) the language network as a whole and (B) each of the six language fROIs. The fROI labels correspond to approximate anatomical locations: IFGorb – the orbital portion of the left inferior frontal gyrus; IFG – left inferior frontal gyrus; MFG – left middle frontal gyrus; AntTemp – left anterior temporal cortex; PostTemp – left posterior temporal cortex; AngG – left angular gyrus. Within each parcel, the responses to the critical experiment conditions are extracted from the top 10% most language-responsive voxels (selected in each of the 21 individuals separately). Error bars indicate standard error of the mean across participants; dots indicate individual participants’ responses.

Although diverse nonlinguistic tasks have been previously shown not to engage the language network (Fedorenko & Varley, 2016), we found here that the language regions responded more strongly during the semantic task on both sentences and pictures compared to the perceptual control task (Figure 2A). A linear mixed effect model with *task* and *stimulus type* as fixed effects and *participant* and *fROI* as random effect intercepts showed a significant effect of task (semantic > perceptual; *β* = 0.93, *p* < 0.001), and stimulus type (sentences > pictures; *β* = 0.23, *p* = 0.018), and an interaction between them (*β* = 0.43, *p* = 0.025). These results demonstrate that the language network responds to the semantic task performed on both sentences and pictures, although this task effect is stronger for sentences.

To investigate individual brain regions comprising the language network, we conducted follow-up analyses on the activity of individual fROIs (FDR-corrected for the number of regions) (Figure 2B). These revealed a significant semantic > perceptual task effect in all fROIs (Table 1). The sentences > pictures stimulus type effect was observed in two fROIs, located in anterior and posterior left temporal lobe. The interaction between task and stimulus type was not significant in any fROI, although, numerically, responses to sentences during the semantic task were stronger than responses to any other condition in all fROIs except the left AngG fROI. We conclude that sensitivity to the semantic task is a general property of all regions in the language network rather than an effect driven by a subset of regions.

To facilitate the comparison of our results with prior neuroimaging studies, we also performed a random effects whole-brain group analysis (see Figure S1 in the online supporting information located at https://www.mitpressjournals.org/doi/suppl/10.1162/nol_a_00030), which yielded results similar to the fROI-based analyses described above. Specifically, we found that the semantic > perceptual contrast for both sentences and pictures activates left-lateralized frontal and temporal regions that overlap with the language parcels (used to constrain the definition of individual language fROIs). The extent of semantics-evoked activation in the left lateral temporal areas was weaker for pictures than sentences (the opposite was true on the ventral surface of the left temporal lobe). Note, however, that these results should be interpreted with caution, since group analyses might conflate functionally distinct regions that are anatomically close (Nieto-Castañón & Fedorenko, 2012), especially in association cortex, which tends to be functionally heterogeneous (Blank et al., 2017; Braga et al., 2019; Fedorenko & Kanwisher, 2009; Frost & Goebel, 2012; Tahmasebi et al., 2012; Vázquez-Rodríguez et al., 2019).

Overall, the first experiment revealed that the language network is strongly and significantly recruited for semantic processing of events presented not only verbally (through sentences), but also nonverbally (through pictures). Specifically, the language network is active when we interpret pictures that depict agent–patient interactions and relate them to stored world knowledge. It is worth noting, however, that responses to the semantic task are stronger for sentences than for pictures (as shown by the interaction between task and stimulus type at the network level; Figure 2A), suggesting that the language network may play a less important role in nonverbal semantic processing. To test whether the engagement of the language network is *necessary* for comprehending visually presented events, we turn to behavioral evidence from individuals with global aphasia.

### Experiment 2: Is the Language Network Required for a Nonverbal Event Semantics Task?

**Figure 3.**
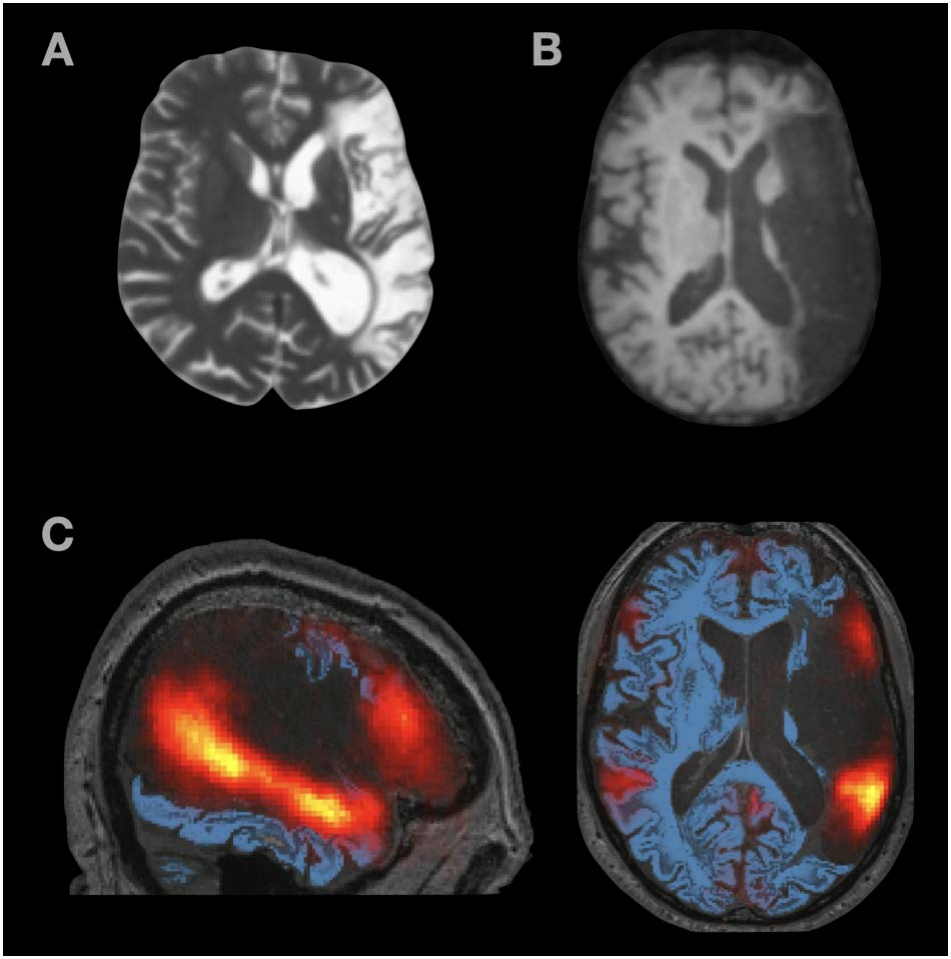
Structural MRI images from (A) S.A. and (B) P.R. (C) Probabilistic language activation overlap map overlaid on top of P.R.’s structural MRI image. The heatmap values range from 0.01 (red) to 0.5 (yellow) and correspond to proportions of individuals (in a set of *n* = 220) that show a significant language localizer (sentences > nonwords) effect in that voxel. As can be seen, the lesion covers most left hemisphere areas with voxels that likely belong to the language network.

We examined two individuals with global aphasia (S.A. and P.R.). Both had suffered large vascular lesions that resulted in extensive damage to left perisylvian cortex, including the language network (see Figure 3 for lesion images, including a probabilistic map of the language network based on fMRI data from neurotypical participants, overlayed onto P.R.’s MRI).

Both individuals were severely agrammatic (Table 2). Whereas they had some residual lexical comprehension ability, scoring well on tasks involving word–picture matching and synonym matching across spoken and written modalities, their lexical production was impaired. Both failed to correctly name a single item in a spoken picture-naming task. S.A. displayed some residual written word production ability, scoring 24 out of 60 in a written picture-naming task. P.R., however, performed poorly in the written task, correctly naming just 2 out of 60 items.

S.A. and P.R.’s syntactic processing was severely disrupted. They scored at or below chance in the reversible spoken and written sentence comprehension tasks (sentence–picture matching), which included active sentences (e.g., “the man kills the lion”), and passive sentences (e.g., “the man is killed by the lion”). They also scored near chance in written grammaticality judgment assessments. The patients’ comprehension performance was impaired regardless of whether the sentences were presented visually or auditorily, indicating that the impairment was linguistic rather than perceptual. To determine whether the sentence comprehension impairments could be explained by working memory deficits, we evaluated the patients’ phonological working memory by means of a digit span test (using a recognition paradigm that did not require language production). The patients’ working memory span was somewhat reduced: S.A. and P.R. had the scores of 3 and 4 items, respectively, compared to the neurotypical age-matched controls who had an average score of 6.4 (*SD* = 0.6; see Zimmerer et al., 2019). However, even such reduced working memory span should have been sufficient for processing the simple subject-verb-object sentences that were used in the syntactic assessments, as well as in the critical task described below. Thus, S.A. and P.R.’s difficulties with linguistic tasks could not be attributed to phonological working memory problems.

Importantly and in line with prior arguments (Fedorenko & Varley, 2016), S.A. and P.R. performed relatively well on nonverbal reasoning tasks, which included measures of fluid intelligence (Raven’s Standard/Colored Progressive Matrices; Raven & Raven, 2003), object semantics (Pyramids and Palm Trees test; Howard & Patterson, 1992), and visual working memory (Visual Pattern Test; Della Sala et al., 1999), indicating that the extensive brain damage in these patients did not ubiquitously affect all cognitive abilities (Table 3). Such a selective impairment of linguistic skills allowed us to examine the causal role of language in nonverbal event semantics.

**Table 3.** Results of nonlinguistic assessments for participants with global aphasia

**Reasoning Tests**	**S.A.**	**P.R.**
Raven’s Colored Progressive Matrices	36/36	34/36
Raven’s Standard Progressive Matrices	53/60	36/60
Pyramids and Palm Trees (3 picture version)	50/52	47/52
Visual Pattern Test	11.5 (90th percentile*)	8.6 (40th percentile*)

*Note*. * Percentiles are calculated with respect to adults in the same age range with no neurological impairment.

To test whether global aphasia affects general event semantics, we measured S.A. and P.R.’s performance on two tasks: (1) the picture plausibility task, identical to the pictures/semantic-task condition from Experiment 1, and (2) a sentence–picture matching task, during which participants saw a picture together with a sentence in which the agent and the patient either matched the picture or were switched (“a cop is arresting a criminal” vs. “a criminal is arresting a cop”); participants had to indicate whether or not the sentence matched the picture. The sentence–picture matching task was similar to the reversible sentence comprehension task in Table 2, except that the pictures were identical to the pictures from the plausibility task and all sentences used active voice. For each task, patient performance was compared with the performance of 12 age-matched controls (58−78 years [mean 65.5 years] for the picture plausibility task; 58−78 years [mean 64.7 years] for the sentence–picture matching task).

**Figure 4.**
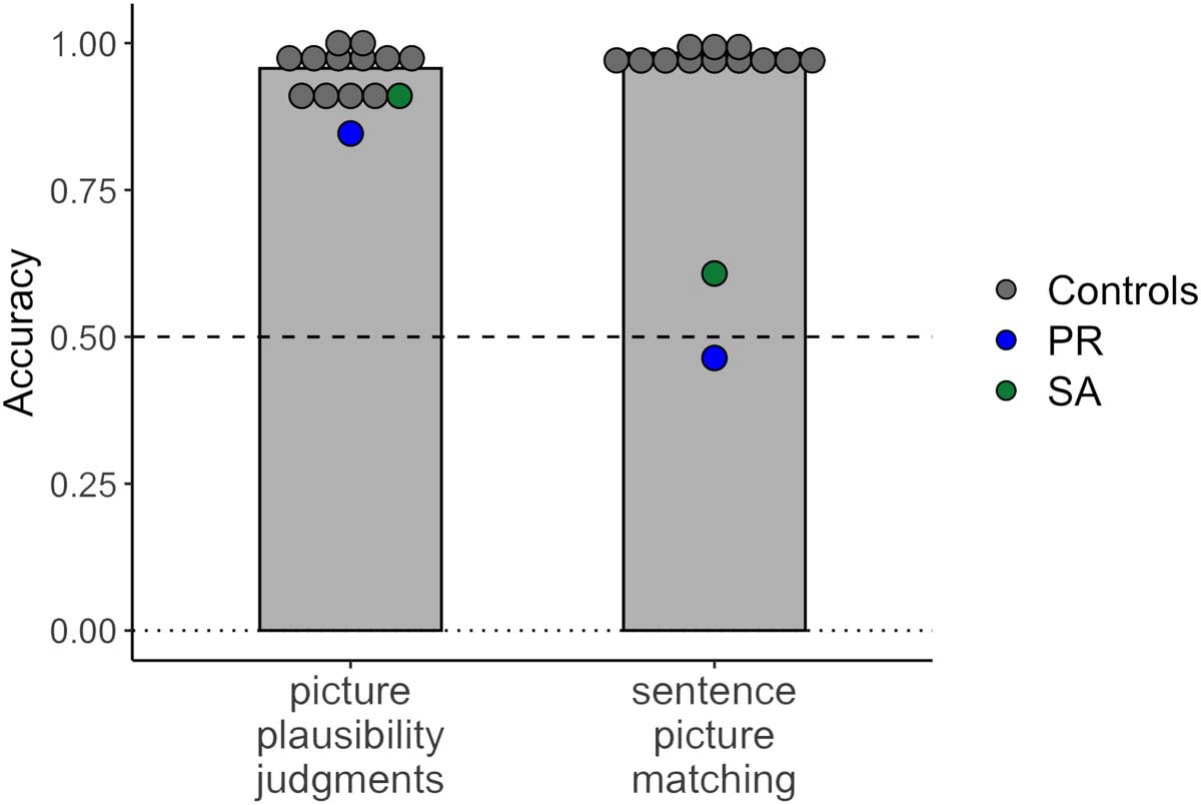
Individuals with profound aphasia perform well on the picture plausibility judgment task but fail on the sentence–picture matching task. Patient accuracies are indicated in blue (PR) and green (SA); average controls’ performance is shown as gray bars; individual controls’ performance (*N* = 12) is shown as gray dots. The dotted line indicates chance performance.

The results showed a clear difference in performance between the picture plausibility task and the sentence–picture matching task (Figure 4), despite the fact that both tasks used the same set of pictures. Both individuals with global aphasia and control participants performed well above chance when judging picture plausibility. Neurotypical controls had a mean accuracy of 95.7% (*SD* = 3.8%). Aphasia patients had mean accuracies of 91.0% (S.A.; 1.2 *SD* below average) and 84.6% (P.R.; 3.0 *SD* below average); the exact binomial test showed that performance of both patients was above chance (S.A., *p* < 0.001, 95% CI [0.82, 0.96]; P.R., *p* < 0.001, 95% CI [0.75, 0.92]). Although their performance was slightly below the level of the controls, the data indicate that both patients were able to process complex semantic (agent–patient) relations to evaluate the plausibility of depicted events.

In the sentence–picture matching task, control participants performed close to ceiling, with a mean accuracy of 98.3% (*SD* = 1.1%). In contrast, both patients were severely impaired: S.A. had a mean accuracy of 60.8% and P.R. had a mean accuracy of 46.4%. The exact binomial test showed that P.R.’s performance was at chance (*p* = 0.464, 95% CI [0.38, 0.55]), while S.A.’s performance was above chance (*p* = 0.009, 95% CI [0.53, 0.69]) but still drastically lower than that of the controls. This result concurs with S.A.’s and P.R.’s poor performance on the reversible sentence comprehension tasks, which had a similar setup but used different materials. However, it stands in stark contrast with the participants’ ability to interpret agent–patient interactions in pictures. The Crawford and Howell (1998) *t* test indicated a significant dissociation between the picture plausibility task and the sentence–picture matching task for both individuals (S.A., *t*(11) = 18.00, *p* < 0.001; P.R., *t*(11) = 24.20, *p* < 0.001). This dissociation held for both hit rate and false alarm rate (Figure S2).

The findings from Experiment 2 demonstrate that, in spite of severe linguistic impairments, individuals with global aphasia were able to access information about event participants depicted in a visual scene, the action taking place between them, the roles they perform in the context of this action, and the real-world plausibility of these roles, indicating that none of these processes require the presence of a functional language network.

## DISCUSSION

The relationship between language and thought has been long debated, both in neuroscience (e.g., Binder & Desai, 2011; Bookheimer, 2002; Fedorenko & Varley, 2016; Friederici, 2020) and other fields (e.g., Carruthers, 2002; Hauser et al., 2002; Vygotsky, 2012; Winograd, 1976). Here, we ask whether language-responsive regions of the brain are essential for a core component of thought: processing combinatorial semantic representations. We demonstrate that left hemisphere language regions are active during the semantic processing of events shown as pictures, although the semantic processing of events shown as sentences elicits a stronger response. We further show that the language network is not essential for nonverbal event semantics, given that the two individuals with global aphasia, who lack most of their left hemisphere language network, can still evaluate the plausibility of visually presented events. Our study advances the field in three ways: (i) it explores relational semantic processing in the domain of events, moving beyond the semantics of single objects—the focus of most prior neuroscience work on conceptual processing; (ii) it evaluates neural overlap between verbal and nonverbal semantics in fMRI at the level of individual participants; and (iii) it provides causal evidence in support of a dissociation between language and nonverbal event semantics. In the remainder of the article, we discuss the implications of our results.

### The Language Network Is Not Required for Nonverbal Event Semantics

Semantic processing of events is a complex, multi-component process. For instance, deciding whether or not an event is plausible requires one to (1) identify the relevant event participants, (2) determine the action taking place between them, (3) decipher the role that each event participant is performing (in our task, agent vs. patient), and finally, (4) estimate the likelihood that a given participant would be the agent/patient of the relevant action. Whereas the first three components can, at least in part, be attributed to input-specific processes (e.g., high-level vision), establishing plausibility cannot be solely attributed to perception: In order to decide whether a cop arresting a criminal is more likely than a criminal arresting a cop, participants need to draw on their world knowledge. We demonstrate that this highly abstract process can proceed even when the language network is severely impaired, thus providing strong evidence that a functional language network is not required for nonverbal semantic processing.

The functional dissociation between language-based and vision-based semantic judgments of events accords with the fact that both non-human animals and preverbal infants are capable of complex event processing (Seed & Tomasello, 2010; Spelke, 1976) and that specialized neural mechanisms, distinct from the language network, have been associated with visual understanding of actions (Fang et al., 2016; Häberling et al., 2016; Tarhan & Konkle, 2020) and interactions between animate and/or inanimate entities (Fischer et al., 2016; Walbrin et al., 2018). These neural mechanisms are either bilateral or right-lateralized, which constitutes further evidence of their dissociation from language, which is typically left-lateralized.

Our results are also consistent with reports of a dissociation between verbal and nonverbal semantic processing of single objects in patients with aphasia (e.g., Antonucci & Reilly, 2008; Bi et al., 2011; Chertkow et al., 1997; Jefferies & Lambon Ralph, 2006; Lambon Ralph et al., 2010) and semantic dementia (e.g., Binney et al., 2016; Gorno-Tempini et al., 2004; Mion et al., 2010; Snowden et al., 2018; Thompson et al., 2003). Those studies typically report that linguistic impairments arise as a result of left hemisphere damage, whereas nonverbal semantic processing deficits are considered to be caused by either bilateral (Lambon Ralph et al., 2017) or right-lateralized lesions (Gainotti, 2011, 2015). Our work contributes to this literature by showing that the language-semantics dissociation holds not only for single concepts but also for combinatorial event-level representations (see also Colvin et al., 2019; Dickey & Warren, 2015). Although we only test two individuals with global aphasia, these data provide an important contribution to the field because of the unique nature of the impairment in these individuals: large-scale disruption of multiple linguistic functions and relatively preserved nonverbal cognition. To test the generalizability of our findings, future work should evaluate a larger sample of individuals with such a dissociation and comprehensively assess both verbal and nonverbal semantic processing of objects, actions, and events.

If language is not essential for event semantics, why is the language network active during a nonverbal event semantics task? It is possible that neurotypical participants partially recode pictorial stimuli into a verbal format (Greene & Fei-Fei, 2014; Trueswell & Papafragou, 2010), which could provide access to linguistic representations as an additional source of task-relevant information (Connell & Lynott, 2013). Indeed, text-based computational models developed in recent years have been shown to successfully perform a wide range of “semantic” tasks, such as inference, paraphrasing, and question answering (Brown et al., 2020; Devlin et al., 2018, among others). Even simple n-gram models can be used to determine the probability of certain events by, for example, estimating the probability that the phrase “is arresting” directly follows “cop” versus “criminal.” Such language-based semantic information is distinct from non-language-based world knowledge (Clark, 2004; Lucy & Gauthier, 2017), and both kinds of information can be flexibly used depending on task demands (Willits et al., 2015). As a result, it is possible that linguistic resources (housed in the language network) provide an additional source of information when neurotypical individuals determine visual *event plausibility*. The absence of this additional information source may account for the small decrement in performance observed in participants with aphasia relative to the control participants.

One might speculate that this “language-based” semantic processing route plays a primary role in neurotypical participants, whereas patients with aphasia rely on some alternative route that arose due to the functional reorganization of the brain postinjury. However, we consider this possibility unlikely. Past behavioral evidence from experiments in neurotypical individuals shows that verbal recoding of visual information is relatively slow and can only occur *after* semantic information has been retrieved from the picture (Potter et al., 1986; Potter & Faulconer, 1975). Furthermore, participants do not typically generate covert verbal labels for visually presented objects unless instructed to do so (Dahan et al., 2001; Magnuson et al., 2003; Rehrig et al., 2020; cf. Meyer et al., 2007) or unless the task imposes memory demands (Pontillo et al., 2015). Our stimuli depicted complex two-participant events, making verbal recoding even more effortful than recoding of single objects and, therefore, unlikely to occur during a task that does not require linguistic label generation (Papafragou et al., 2008). Finally, even if individuals with aphasia did rely on a compensatory (e.g., right hemisphere mediated) mechanism for semantic processing, it would still indicate that brain mechanisms outside of the core left hemisphere language network are capable of supporting combinatorial semantics, thus underscoring our claim that language and nonverbal event semantics are neurally dissociable.

Future work should further investigate the nature of the language network’s responses to nonverbal stimuli. Although some studies, like ours, have reported that the left hemisphere language regions have stronger responses to sentences than to content-matched pictures (Amit et al., 2017), others have reported the opposite preference (Jouen et al., 2015). The divergent result in Jouen et al. (2015) is most likely due to differences in the analytic approach, namely, in the use of ROIs derived from group analyses as opposed to functionally defined fROIs. Task demands could also contribute to the difference in results: Jouen et al. used a one-back memory task (no condition-specific behavioral results were reported), whereas we used a plausibility judgment task that had similar accuracies and reaction times between the sentences and pictures. The fact that we found an interaction between input type (sentences vs. pictures) and task also indicates that task effects on activity in the language network merit additional investigation (although see Cheung et al., 2020, for evidence that task demands often have little effect on the responses of the language regions to verbal stimuli). The task effects observed in our study cannot be explained by task difficulty: The participants’ accuracies for the semantic versus perceptual task were not significantly different; the reaction times were slightly faster for the perceptual task, but the effect size was small (0.06 s, with average trial RT = 1.21 s) and therefore unlikely to fully account for the neural effect. Moreover, the language network is not generally driven by task difficulty (Diachek et al., 2020) and shows strong, consistent responses even in the absence of task (Baldassano et al., 2018; Brennan et al., 2016; Huth et al., 2016; Scott et al., 2017; Shain et al., 2020; Wehbe et al., 2014, among others). Thus, future work needs to explore the effects of task content rather than task difficulty per se.

### Implications for Theories of Semantics in the Brain

In this paper, we focused on the *role of the language network* in nonverbal event semantics, not on the question of which cognitive and neural mechanisms support modality-invariant event processing (we report those analyses in other work; Ivanova et al., 2021). Nonetheless, current results also bear on general theories of semantic processing in the mind and brain.

Many current theories of semantics highlight broad anatomical areas implicated in linguistic processing as putative semantic hubs. Those include left AngG (e.g., Binder & Desai, 2011), left inferior frontal cortex (e.g., Hagoort & van Berkum, 2007), and the anterior temporal lobes (ATL; e.g., Patterson et al., 2007). However, the areas in question are large patches of cortex that are structurally and functionally heterogeneous: As a result, simply because a visual-semantics study reports activation within the left IFG or AngG does not mean that the *language-responsive* portions of those broad areas are at play (see, e.g., Fedorenko & Blank, 2020, for discussion).

In the current study, the language-responsive fROIs that we defined within left AngG, left inferior frontal cortex, and left ATL all responded more strongly during the semantic task than during the perceptual task, for both sentences and pictures. Although this pattern is consistent with evidence of their general involvement in semantic processing, it goes against some of the specific claims made in the literature. For example, our results are inconsistent with the claim that the angular gyrus is the *primary* region involved in event semantics (Binder & Desai, 2011; cf. Williams et al., 2017) given that other regions show a similar functional response profile. That said, the fROI in the angular gyrus was the only one that showed numerically stronger responses to pictures than to sentences, consistent with evidence of its involvement in processing (at least some) semantically meaningful nonverbal stimuli (Amit et al., 2017; Baldassano et al., 2017; Fairhall & Caramazza, 2013; Handjaras et al., 2017; Pritchett et al., 2018). Our results also provide some evidence that a portion of the left ATL is engaged in processing event-level representations in verbal stimuli (Jackson et al., 2015; Teige et al., 2019; cf. Lewis et al., 2015; Schwartz et al., 2011; Xu et al., 2018, who claim that the ATL is involved in retrieving property-level but not event-level information). Finally, we observed that the ATL language fROI responded more strongly to sentences than to pictures, which might speak against its role as an amodal semantic hub. Note, however, that this fROI encompasses only a small fraction of left ATL; it therefore remains possible that some other parts of the ATL—especially its ventral/ventromedial portions—have a modality-invariant response profile (Lambon Ralph et al., 2017; Visser et al., 2012).

In addition, our findings contribute to the body of work on the neural representation of agent–patient relationships. Previous experiments attempting to localize brain regions that support thematic role processing have attributed the processing of agent–patient relations to the left hemisphere. Frankland and Greene (2015, 2020) used sentence stimuli to isolate distinct areas in left superior temporal sulcus (STS) that are sensitive to the identity of the agent versus the patient. J. Wang et al. (2016) found that the same (or nearby) STS regions also contained information about thematic roles in videos depicting agent–patient interactions. However, the latter study identified a number of other regions that were sensitive to thematic role information, including clusters in right posterior middle temporal gyrus and right angular gyrus, suggesting that left STS is not the only region implicated in thematic role processing. A similar distributed pattern was also reported in a neuropsychological study (Wu et al., 2007) that found that lesions to mid-STS led to difficulties in extracting thematic role information from both sentences and pictures; however, deficits in visual agent–patient processing were additionally associated with lesions in anterior superior temporal gyrus, supramarginal gyrus, and inferior frontal cortex, which casts further doubt on the unique role of the left STS in agent–patient relation processing. In sum, the evidence to date suggests that parts of the left STS may play a role in processing linguistic information, including thematic relations (Frankland & Greene, 2015, 2020) and verb argument structure (Elli et al., 2019; Williams et al., 2017), but additional brain regions support the processing of event participant roles in nonverbal stimuli.

Finally, our results are generally consistent with a distributed view of semantic representations (McClelland & Rogers, 2003; Tyler & Moss, 2001). Multiple recent studies found that semantic information is not uniquely localized to any given brain region but rather distributed across the cortex (e.g., Anderson et al., 2017; Huth et al., 2016; Pereira et al., 2018; X. Wang et al., 2018). Distributing information across a network of regions in both left and right hemispheres enables the information to be preserved in case of brain damage (Schapiro et al., 2013), which would explain why patients with global aphasia preserve the ability to interpret visually presented events. That said, the findings reported here do not speak to the question of whether such representations rely primarily on sensorimotor areas (Barsalou, 2008; Pulvermuller, 1999) or on associative areas (Mahon, 2015; Mahon & Caramazza, 2008).

### Implications for Neuroimaging Studies of Amodal Semantics

The non-causal nature of the language network activation during a nonverbal semantic task has important implications for the study of amodal/multimodal concept representations. A significant body of work has aimed to isolate amodal representations of concepts by investigating the overlap between regions active during the viewing of verbal and nonverbal stimuli (Bright et al., 2004; Devereux et al., 2013; Fairhall & Caramazza, 2013; Handjaras et al., 2017; Sevostianov et al., 2002; Thierry & Price, 2006; Vandenberghe et al., 1996; Visser et al., 2012; A. D. Wagner et al., 1997). Most of these overlap-based studies have attributed semantic processing to frontal, temporal, and/or parietal regions within the left hemisphere. Our work, however, demonstrates that, even though meaningful linguistic and visual stimuli evoke overlapping activity in left-lateralized frontal and temporal regions, conceptual information about events persists even when most of these regions are damaged. Thus, overlapping areas of activation for verbal and nonverbal semantic tasks observed in brain imaging studies do not necessarily play a causal role in amodal event semantics.

Overall, our study emphasizes the importance of investigating combinatorial semantic processing using both verbal and nonverbal stimuli. Our results show that semantic processing of visually presented events does not require the language network, drawing a sharp distinction between language and nonverbal event semantics and highlighting the necessity to characterize the relationship between them in greater detail using a combination of brain imaging and patient evidence.

## ACKNOWLEDGMENTS

We would like to acknowledge the Athinoula A. Martinos Imaging Center at the McGovern Institute for Brain Research at MIT, and its support team (Steve Shannon and Atsushi Takahashi). We thank Birgit Zimmerer for creating the picture stimuli used in both experiments, Chloe Bustin for norming the stimuli, Lily Jordan for help with the behavioral piloting of the fMRI experiment, and EvLab members for their help with fMRI data collection. Evelina Fedorenko was supported by NIH awards R00-HD057522, R01-DC016607, and R01-DC016950, by a grant from the Simons Foundation to the Simons Center for the Social Brain at MIT, and by funds from BCS and the McGovern Institute for Brain Research at MIT. Rosemary Varley was supported by Arts and Humanities Research Council and Alzheimer’s Society awards.

## FUNDING INFORMATION

Evelina Fedorenko, National Institutes of Health (http://dx.doi.org/10.13039/100000002), Award ID: R00-HD057522. Evelina Fedorenko, National Institutes of Health (http://dx.doi.org/10.13039/100000002), Award ID: R01-DC016607. Evelina Fedorenko, National Institutes of Health (http://dx.doi.org/10.13039/100000002), Award ID: R01-DC016950. Evelina Fedorenko, Simons Foundation (http://dx.doi.org/10.13039/100000893). Evelina Fedorenko, McGovern Institute for Brain Research at MIT. Evelina Fedorenko, Massachusetts Institute of Technology (http://dx.doi.org/10.13039/100006919). Rosemary Varley, Arts and Humanities Research Council (http://dx.doi.org/10.13039/501100000267). Rosemary Varley, Alzheimer’s Society.

## AUTHOR CONTRIBUTIONS

**Anna Ivanova:** Data curation; Formal analysis; Investigation; Software; Validation; Visualization; Writing – original draft: preparation. **Zachary Mineroff:** Data curation; Formal analysis; Investigation; Software. **Vitor Zimmerer:** Conceptualization; Data curation; Investigation; Methodology; Writing – review & editing. **Nancy Kanwisher:** Conceptualization; Supervision, Writing – review & editing. **Rosemary Varley:** Conceptualization; Funding acquisition; Methodology; Project administration; Resources; Supervision; Writing – review & editing. **Evelina Fedorenko:** Conceptualization; Funding acquisition; Methodology; Project administration; Resources; Supervision; Writing – review & editing.

## TECHNICAL TERMS

Semantic knowledge: Generalized, abstract information about objects, properties, scenes, actions, and ideas.
Semantic processing: The process of accessing and manipulating semantic knowledge.
The language network: A set of left-lateralized regions in the frontal and temporal lobes that show selective responses to spoken and written language.
Global aphasia: A severe form of language impairment caused by damage to the language network, resulting in substantial impairments in both production and comprehension.
Event: An action along with the entities participating in that action.
Thematic role: The role that a given entity plays in an event, such as agent or patient.
Agent: The event participant administering the action.
Patient: The event participant that is being acted upon.
Event plausibility: The likelihood of a given event happening in the real world.
