# Supplementary material for: The Language Network Is Recruited but Not Required for Nonverbal Event Semantics
Source: Neurobiol Lang (Camb). 2021 Mar 17;2(2):176–201. doi: 10.1162/nol_a_00030 (PMC10158592; doi:10.1162/nol_a_00030)
Supplement: Supplementary file 1 [file nol-2-2-176-s001.docx]

**The language network is recruited but not required
for nonverbal event semantics**

**Supplemental Information**

**Experiment 1**


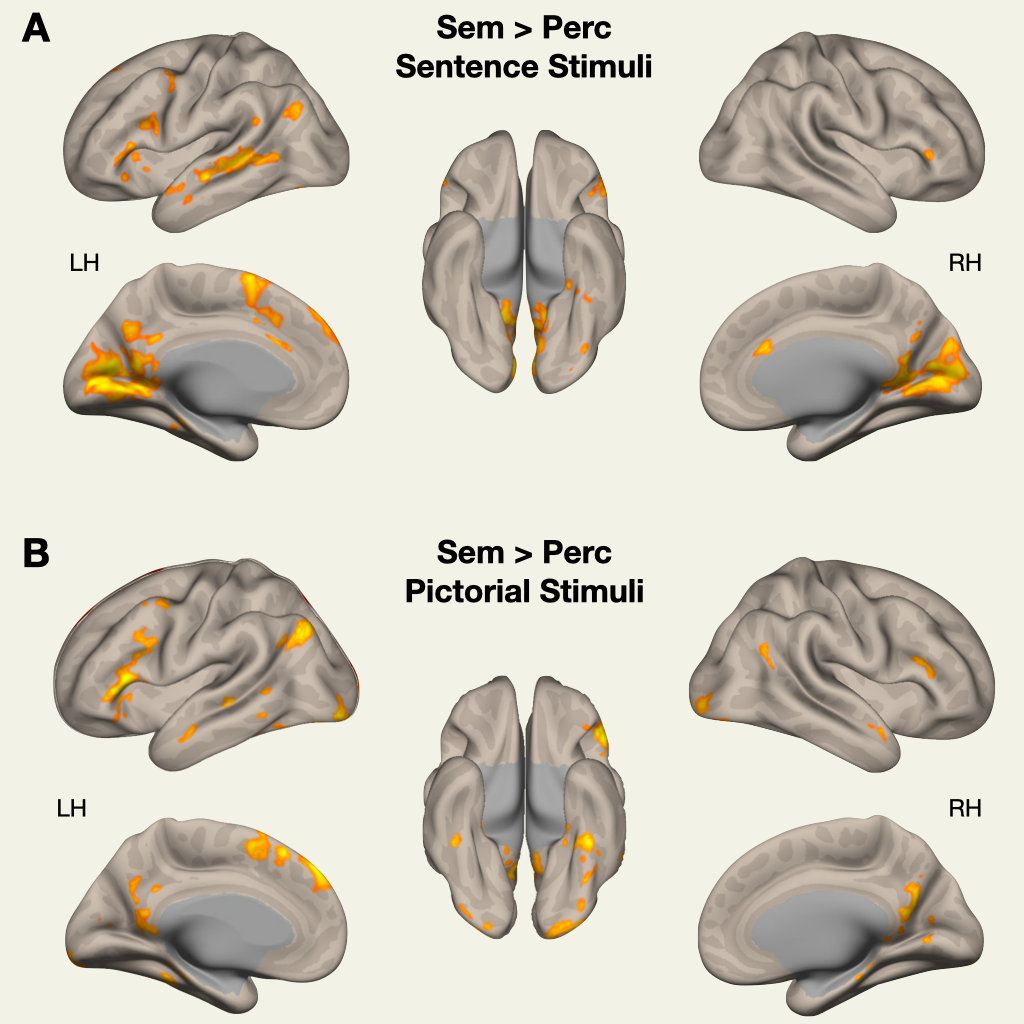


***Figure S1.*** *Whole-brain random effects group analysis (Holmes&Friston, 1998) for Semantic > Perceptual task contrast, conducted separately on the data from sentence trials (a) and picture trials (b). The analysis was conducted using the spm_ss toolbox (available at http://www.nitrc.org/projects/spm_ss), which interfaces with SPM and the CONN toolbox (https://www.nitrc.org/projects/conn). The results were thresholded at p=0.001, and resulting clusters were FDR-corrected at p=0.05.*

**Experiment 2**

**
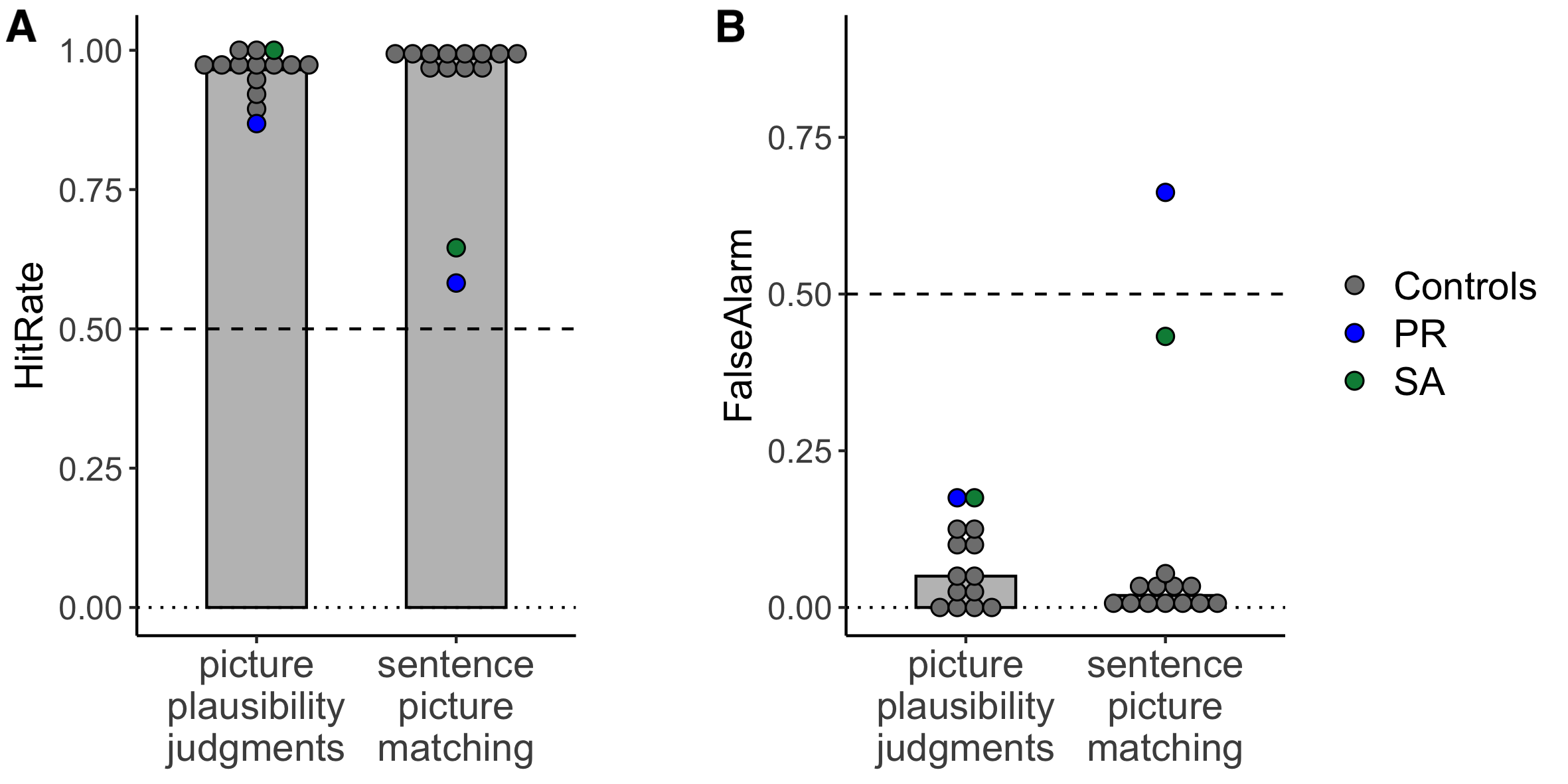
**

***Figure S2.*** *Hit rate (A) and false alarm rate (B) for Experiment 2 tests. Error bars indicate standard error of the mean. The Crawford-Howell test indicated a significant dissociation between the two tests for both hit rate (S.A.: t(11) = 18.95, p < .001; P.R.: t(11) = 19.59, p < .001) and false alarm rate (S.A.: t(11) = 12.55, p < .001; P.R.: t(11) = 20.31, p < .001).*

**References**

Holmes, A. P., & Friston, K. J. (1998). Generalisability, Random Effects & Population Inference. *NeuroImage*. https://doi.org/10.1016/S1053-8119(18)31587-8
